# Application of machine learning to nanomaterial design with silver nanoprisms

**DOI:** 10.1186/s11671-026-04503-y

**Published:** 2026-03-11

**Authors:** Constantin Richard, Paul-Adrien Pichon, Jaroslava Nováková, Neda Irannejad Najafabadi, Jacinto Sá

**Affiliations:** 1 Department of Chemistry-Ångström, Physical Chemistry Division, Uppsala University, 751 20 Uppsala, Sweden; 2 Chimie ParisTech, Université Paris Sciences et Lettres (PSL), Paris, 75005 France; 3 Department of Surface and Plasma Science, Charles University, 18000 Prague 8, Czechia; 4 Institute of Physical Chemistry, Polish Academy of Sciences, 01-224 Warsaw, Poland

**Keywords:** Machine learning in nanomaterials synthesis, Convolutional neural networks (CNNs), Silver nanoprisms, In situ spectroscopy, Predictive materials design

## Abstract

Achieving reproducible synthesis of nanomaterials with tunable optical and morphological properties remains a central challenge in materials design. Conventional trial-and-error strategies struggle with the nonlinear transitions governing nanoparticle growth, often limiting control over plasmonic responses. Here, we introduce a convolutional neural network (CNN) framework that couples in situ time-resolved UV–Vis spectroscopy with the synthesis of silver nanoprisms, extracting predictive rules for morphology and optical behavior. By leveraging transient spectral dynamics rather than endpoint data alone, the model captures hidden growth pathways and accurately predicts final size distributions and plasmonic signatures from a modest experimental dataset. This machine-learning-assisted methodology integrates directly into synthesis workflows, reducing experimental burden while enhancing reproducibility. Beyond silver nanoprisms, the strategy provides a transferable route for rational design of nanomaterials with tailored optical functionalities, advancing the broader goal of data-driven materials design.

## Introduction

The discovery of advanced nanomaterials with tailored properties is central to addressing urgent challenges in sustainable energy conversion, storage, and efficiency. Applications such as photocatalysis for solar H_2_ [1, 2], photovoltaics [3], plasmon-enhanced electrocatalysis [4, 5], and smart thermal coatings [6, 7] rely critically on the ability to design materials whose morphology and optical properties can be finely tuned to optimize performance [8]. Yet, despite decades of progress, reproducible and scalable synthesis remains a key bottleneck, motivating continuous-flow platforms with in-line analytics and strict impurity control [9, 10]. Traditional optimization strategies often fail to capture the complexity of nanomaterial growth, where small perturbations in reagent concentration, mixing rate, or reaction environment can lead to large morphological shifts and, in turn, drastic changes in functionality.

Silver nanoprisms exemplify this broader challenge. Their strong and tunable surface plasmon resonances span the visible to near-infrared region, making them highly relevant for light-harvesting, sensing, and thermal management applications [11]-17]. Yet, despite significant advances in synthetic chemistry, the reproducible scale-up of such nanostructures remains a major bottleneck for industrial translation [[18]]. The emergence of the *Nanomaterials-as-a-Service* (NaaS) paradigm, where users request materials with targeted functionalities, underscores the need for robust, flexible, and automated synthesis strategies [19]. Achieving this goal requires continuous-flow systems capable of real-time monitoring and adaptive control. However, conventional optimization frameworks such as Design of Experiments or Bayesian optimization [19, 20] often fail in this context, as they rely on smooth response surfaces and low-noise outputs. In practice, nanomaterial synthesis involves abrupt morphological transitions, stochastic nucleation events, and spectroscopic noise, all of which obscure meaningful trends and hinder reproducibility.

Recent advances in machine learning (ML) provide powerful tools for addressing the complexity of nanomaterial synthesis, where nonlinear growth dynamics, stochastic nucleation events, and spectroscopic noise often limit the effectiveness of conventional optimization strategies. In particular, convolutional neural networks (CNNs) excel at capturing nonlinear relationships in high-dimensional datasets without requiring explicit mechanistic models. For example, Bilén et al. [21] demonstrated that CNN-based models can accurately infer average size and polydispersity of gold nanoparticles from in silico UV–Vis spectra, even in the presence of spectral noise.

Beyond spectral interpretation, ML frameworks have also been applied to actively guide synthesis. Notably, the two-step Bayesian optimization combined with neural-network regression developed by Mekki-Berrada et al. [22] enables optimization of nanoparticle synthesis toward a target optical signature using a limited number of experiments, illustrating the power of inverse-design strategies. More broadly, the rapidly expanding literature on machine learning for nanomaterial synthesis, characterization, and functional property prediction, surveyed in the recent review by Diao et al. [23], highlights how CNNs and related AI methods can accelerate materials discovery for energy-related applications.

While these approaches have proven highly effective, they typically rely on endpoint measurements and assume relatively smooth response surfaces in parameter space. In contrast, nanomaterial growth often proceeds through transient, non-monotonic pathways that are not fully captured by final-state descriptors alone. In the present work, we focus on embedding *transient, *in situ spectroscopic information directly into the learning process. By leveraging the temporal evolution of UV–Vis spectra during nanoparticle growth, the CNN/LSTM architecture captures dynamic features that are inaccessible to endpoint-only models or parameter-space optimization methods.

This strategy is particularly well suited for protocol refinement and scale-up, where reproducibility and sensitivity to subtle changes in growth pathways are critical. Importantly, the proposed approach is not intended to replace mechanistic modeling or global optimization frameworks such as Bayesian optimization, but rather to complement them by providing a data-efficient route for learning synthesis–structure–property relationships from experimentally accessible, time-resolved data.

Here, we demonstrate a CNN-assisted framework for the discovery and refinement of synthesis protocols using silver nanoprisms as a model system. By systematically varying experimental conditions and incorporating in situ transient UV–Vis spectroscopic data collected during nanoparticle growth, we establish a predictive workflow that links synthesis parameters to nanoparticle morphology and optical properties. Beyond silver nanoprisms, this strategy offers a transferable pathway for rational nanomaterial design, with potential relevance for energy conversion, catalysis, and photonic applications.

## Experimental details

### Synthesis of silver nanoparticles

The synthesis was carried out following the seed-mediated growth protocol originally reported by Aherne et al. [11], with the only modification being the final stabilization step. In our procedure, polyvinylpyrrolidone (PVP) was added after nanoparticle growth to encapsulate and stabilize the colloids, replacing citrate. During the initial nucleation step, small atomic or molecular clusters, commonly referred to as *seeds*, form once the precursor concentration exceeds a critical supersaturation. These seeds serve as the initial nuclei for particle growth, and subsequent addition of atoms or ions results in the controlled development of nanoparticles toward their final size and morphology. The seed volume added in the growth step affects nucleation density and size distribution. The protocol consists of three steps, summarized below:

*Seed preparation:* Silver nanoparticle seeds were synthesized following Aherne et al. [11] established protocol.Briefly, seeds were generated in a three-neck flask by combining 40 mL of trisodium citrate solution with 2 mL of polystyrene sulfonate (PSSS). Under vigorous stirring, 2.4 mL of freshly prepared NaBH_4_ solution was introduced, followed by the controlled addition of AgNO_3_ solution (0.5 mM, 2 mL/min for 2 min). The formation of a pale-yellow dispersion confirmed seed formation.

*Growth step:* In a separate three-neck flask, 40 mL of deionized water was mixed with 600 µL of ascorbic acid solution and 60 or 70 µL of the seed dispersion. AgNO_3_ solution (0.5 mM) was added continuously at 1 mL/min under stirring. During this stage, the reaction mixture was circulated through a UV–Vis flow cell (2 mL/min) to enable real-time spectral monitoring for 14 min.

*Stabilization:* After completion of the AgNO_3_ addition, 2 mL of PVP (Mw 40′000, 6.25 mg in 25 mL water) solution was introduced to stabilize the nanoprisms and prevent aggregation.

*Experimental variables for CNN protocol discovery*: To evaluate the ability of a CNN to identify effective synthesis conditions, we systematically varied the citrate concentration during seed synthesis (30–50 mL). This parameter strongly influenced seed quality and, in turn, the morphology of the resulting nanoparticles. The seeds obtained under these conditions were then used in the growth step, with two different initial seed volumes (60 or 70 µL). These controlled variations generated a diverse dataset of nanoparticle shapes and optical signatures, which was subsequently used to train and validate the CNN model.

### Silver nanoparticles characterisation

Nanoparticles obtained under optimized conditions were characterized by UV–Vis spectroscopy, dynamic light scattering (DLS), and transmission electron microscopy (TEM). DLS measurements were performed with a Malvern Zetasizer Nano ZS, and in-flow UV–Vis spectra were acquired using an Ocean Insights Flame spectrophotometer equipped with a Z-cell and fiber optics. Morphology and structural features were examined by high-resolution TEM (HRTEM) on a JEOL JEM-2200FS microscope operated at 200 kV, offering a point resolution of 0.19 nm and a lattice resolution of 0.10 nm. TEM samples were prepared by depositing a droplet of the aqueous nanoparticle dispersion onto a 300-mesh copper grid coated with a lacey carbon film (Agar Scientific). Acquired images were processed and analyzed using the Digital Micrograph software package.

### Machine learning motivation and model architecture

A central limitation of machine learning (ML) methods in materials discovery is the requirement for large, high-quality datasets. In practice, such datasets must often be generated experimentally, since synthetic or simulated data typically lack the variability and noise characteristics necessary to train robust predictive models. This reliance on experimental data makes ML adoption in nanomaterials research both time-consuming and resource-intensive, often limiting throughput and constraining hardware availability.

To address this challenge, we hypothesized that the number of experiments required for model training could be substantially reduced by leveraging in situ transient spectroscopic information. Specifically, a CNN was trained on time-evolving UV–Vis spectra collected during nanoparticle synthesis, together with the corresponding reaction conditions. By exploiting the temporal dynamics of spectral evolution, the model can extract physically meaningful latent representations that accelerate learning and reduce the data burden.

**Fig. 1:**
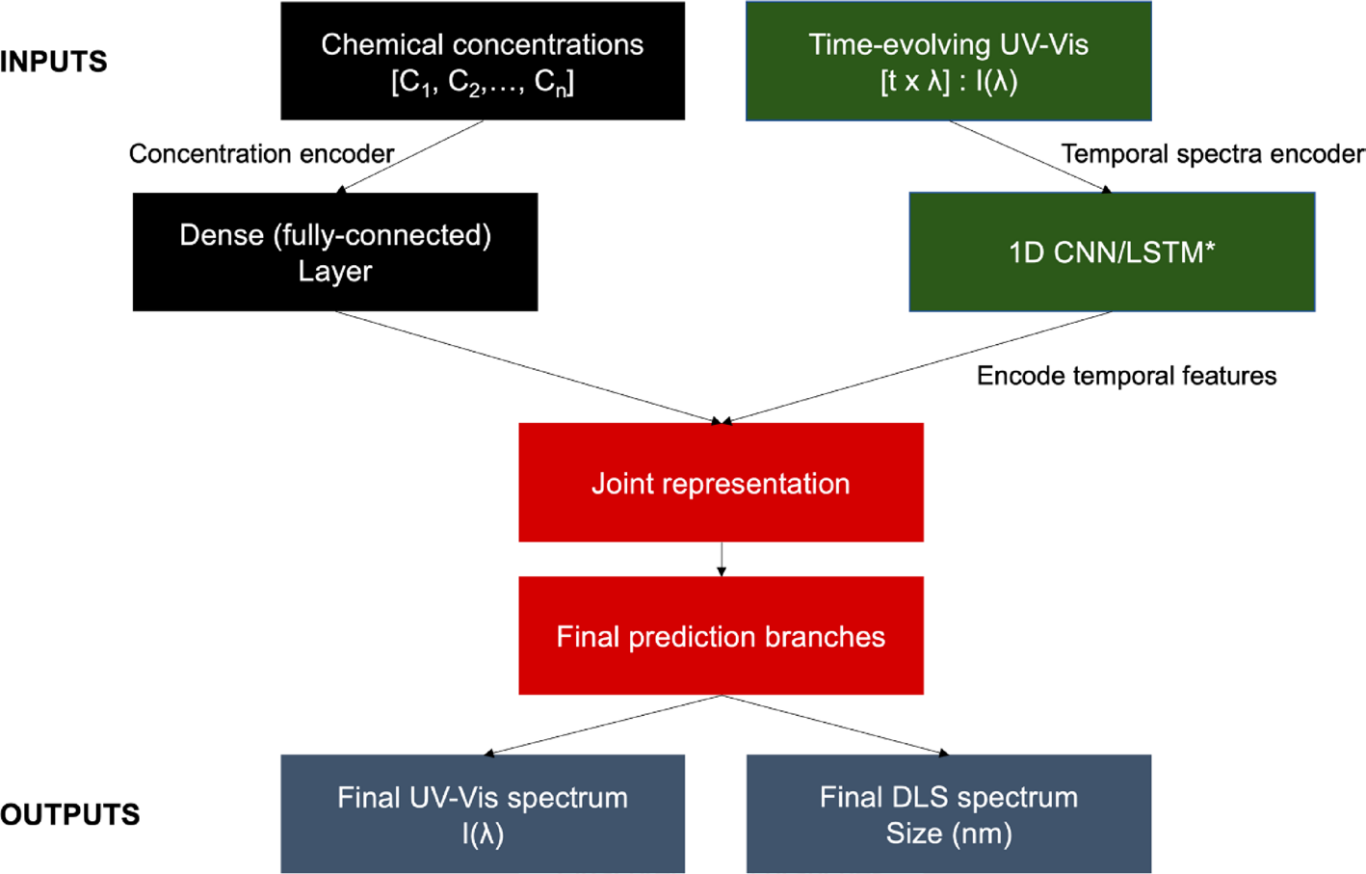
Schematic of the machine learning architecture used for protocol discovery. Experimental inputs consist of reagent concentrations and in situ time-resolved UV–Vis spectra. Spectral data are processed through a 1D CNN/LSTM module, where convolutional filters capture local spectral features (e.g., peak position and shape) and recurrent layers encode their temporal evolution during nanoparticle growth. These spectral–temporal embeddings are fused with the concentration branch in a joint latent space, which is used to predict the final optical spectrum and size distribution of the nanoparticles. This design enables the model to learn growth dynamics from a reduced number of experiments while maintaining predictive accuracy. *1D CNN/LSTM module is explained below

The architecture, shown schematically in Fig. 1, employs a teacher–student/self-supervised strategy in which two complementary input streams are jointly encoded. The first input comprises the experimental synthesis parameters, represented by the citrate volume used during seed formation and the seed volume employed in the growth step, encoded as a numerical feature vector. The second input consists of time-resolved UV–Vis spectra acquired *i*n situ during nanoparticle growth, represented as a two-dimensional time–wavelength intensity matrix. Prior to training, spectral data were normalized to account for absolute intensity variations while preserving relative peak evolution and spectral shape.

The network was trained in a supervised manner to predict two experimentally measurable outputs: (a) the final UV–Vis spectrum of the nanoparticles and (b) the DLS size distribution capturing the final particle size distribution. During training, the transient UV–Vis time series acted as auxiliary supervision, guiding the CNN to learn meaningful growth dynamics. For each prediction task, one experiment was selected as the target output, while the remaining experiments within the same synthesis regime were used for training. Model performance was evaluated by comparing predicted and experimental outputs using established metrics, including MAE (Mean Absolute Error), RMSE (Root Mean Squared Error), and R^2^ (Coefficient of Determination) for DLS predictions, and Cosine similarity and Pearson correlation for UV–Vis spectra.

While formal k-fold or leave-one-out cross-validation (LOOCV) is standard practice for large machine-learning datasets, these approaches have known limitations when applied to very small experimental datasets. In materials science, cross-validation (including LOOCV) is commonly used to estimate model generalization, but its interpretation can be challenging in data-scarce regimes where withholding even a single sample significantly reduces the available training set and can introduce distributional bias in performance estimates [24-26].

Owing to the intentionally limited size of the experimental dataset, the validation strategy focuses on assessing predictive consistency within closely related synthesis conditions rather than establishing broad statistical generalizability. The present work therefore aims to demonstrate data efficiency and feasibility rather than universal predictive coverage.

This hybrid strategy enables the CNN to fuse experimental concentrations with spectral-temporal information, allowing it to build an internal representation of nanoparticle growth while avoiding the prohibitive need for large experimental datasets.

The objective of the present work is not to benchmark predictive performance across different model classes, but to demonstrate the added value of embedding transient, in situ spectroscopic information into synthesis workflows. Models trained solely on endpoint spectra or static synthesis parameters cannot exploit growth dynamics and therefore address a fundamentally different prediction problem. For this reason, systematic baseline comparisons and ablation studies are deferred to future work, where larger datasets will enable fair and statistically meaningful benchmarking.

The 1D CNN/LSTM module was designed to capture both local spectral features and their temporal evolution during nanoparticle growth. The one-dimensional convolutional layers apply sliding filters along the wavelength axis to detect peak positions, shapes, and intensity changes in the UV–Vis spectra, producing compact feature representations that are robust to spectral noise and minor shifts. These sequential features are then passed to a long short-term memory (LSTM) network, which encodes temporal dependencies through gated memory cells. In this way, the combined CNN/LSTM architecture extracts physically meaningful embeddings that reflect both instantaneous spectral characteristics and longer-term dynamic trends.

Key architectural and training parameters, including convolutional filter sizes, number of LSTM units, learning rate, and optimization settings, were fixed across all experiments to ensure consistency and reproducibility.

## Results and discussion

**Fig. 2:**
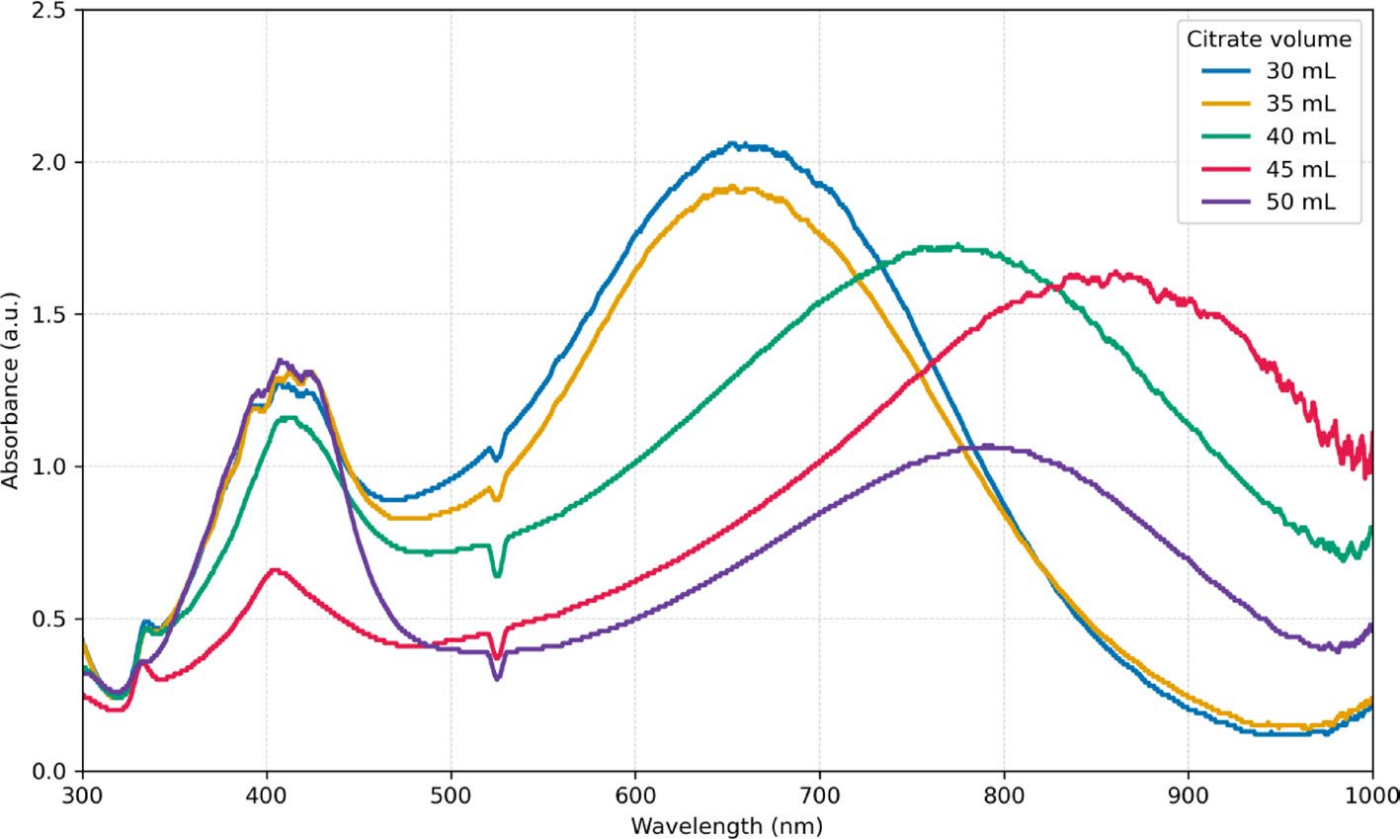
UV–Vis spectra of silver nanoprisms synthesized with 70 µL of seed solution. Seeds were generated using different citrate concentrations during the seed formation step, resulting in variations in the final optical response

**Fig. 3:**
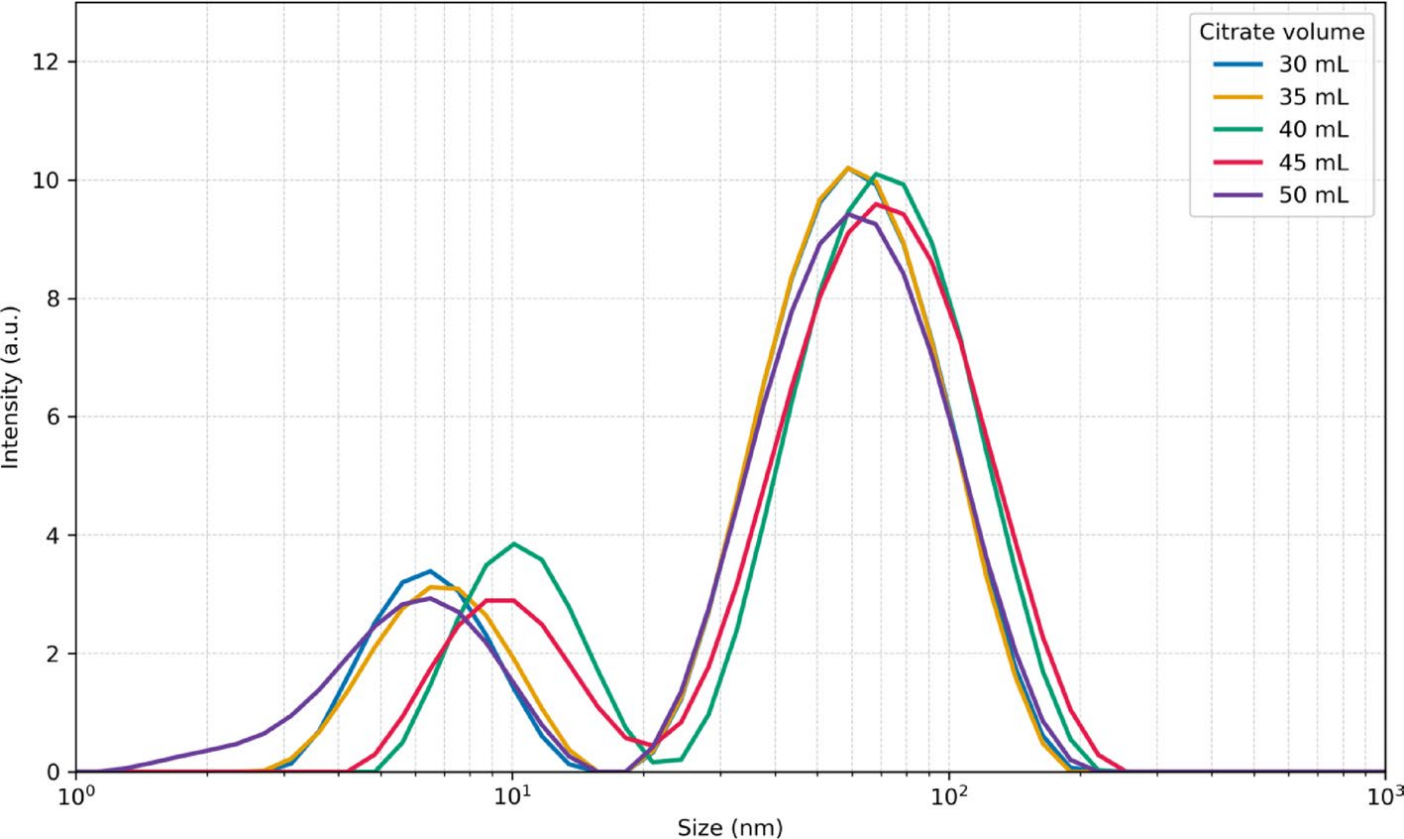
DLS size distributions of silver nanoprisms synthesized with 70 µL of seed solution. Seeds were generated using different citrate concentrations during the seed formation step, leading to distinct hydrodynamic size populations

The volume of seed solution added during the growth step plays a critical role in determining nucleation density and, consequently, the size distribution of the resulting nanoparticles. To evaluate this effect within our approach, particles were synthesized using two different seed volumes (60 and 70 µL). These seeds were generated in the initial seed formation step by varying the citrate concentration (30–50 mL), thereby producing seeds of different quality. Figures 2 and 3 show the final UV–Vis spectra and DLS size distributions of nanoparticles obtained with 70 µL of seed solution. These datasets were used as target outputs to train the CNN model and to benchmark its predictive performance.

**Fig. 4:**
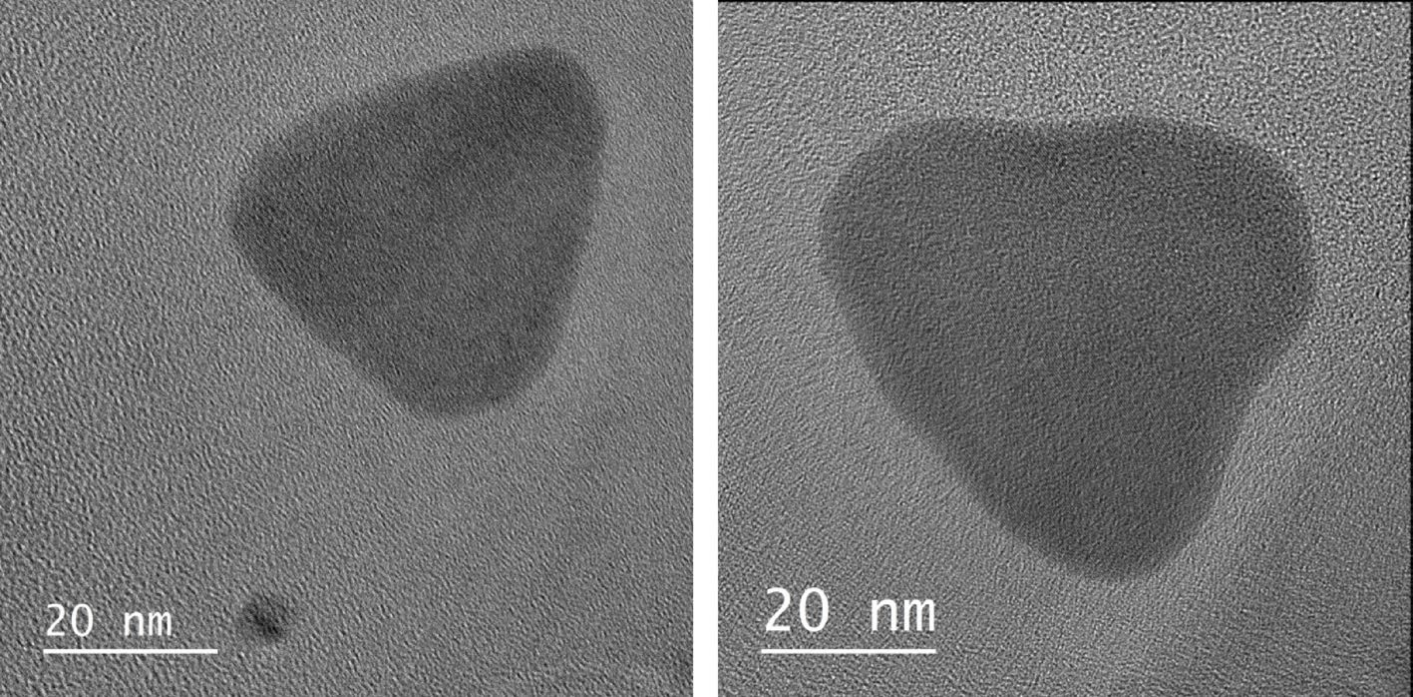
TEM images of silver nanoprisms synthesized using 70 µL of seed solution prepared with 40 mL of citrate. Two representative particles from the same batch are shown, highlighting the triangular morphology and the thin plate-like structure characteristic of nanoprisms

The sample prepared with 40 mL of citrate during seed synthesis (green trace in Figs. 2 and 3) corresponds to a scaled-up version of the Aherne et al. protocol [11], where all reagents were increased eightfold to yield a larger final volume of nanoparticles. The UV–Vis spectrum of this sample displays the two characteristic plasmonic features of thin silver nanoprisms: a peak at 410 nm associated with the out-of-plane (thickness) resonance and a dominant in-plane resonance centered at 750 nm. The corresponding DLS profile exhibits two distinct size populations, centered at approximately 10 nm and 65 nm. TEM analysis (Fig. 4) confirms the expected triangular plate-like morphology with edge lengths in the range of 45–55 nm, and the relatively low contrast in the particle core is consistent with a plate thickness below 10 nm.

A direct comparison between DLS and TEM highlights the complementary nature of these techniques for characterizing anisotropic silver nanoprisms. TEM provides direct visualization of particle morphology and lateral dimensions, whereas DLS measures the hydrodynamic size of particles in solution, which includes contributions from the stabilizing PVP shell and solvation layer and therefore yields systematically larger apparent dimensions. While standard DLS reports an averaged hydrodynamic size and does not explicitly resolve individual particle axes, anisotropic plate-like nanoparticles can give rise to multimodal size distributions. In the present case, the two DLS populations are consistently observed and, based on direct comparison with TEM, are attributed to the effective hydrodynamic thickness (≈5–10 nm) and lateral dimensions (≈65–80 nm) of the nanoprisms. The slight offset between TEM and DLS values is expected and arises from polymer capping and hydration effects.

Importantly, DLS is not used here as a substitute for direct morphological characterization but rather as a reproducible, solution-phase descriptor of nanoparticle size distributions. Given that DLS-derived distributions are one of the target outputs for the machine-learning model, their use is both appropriate and sufficient for evaluating predictive performance within the scope of this study.

**Fig. 5:**
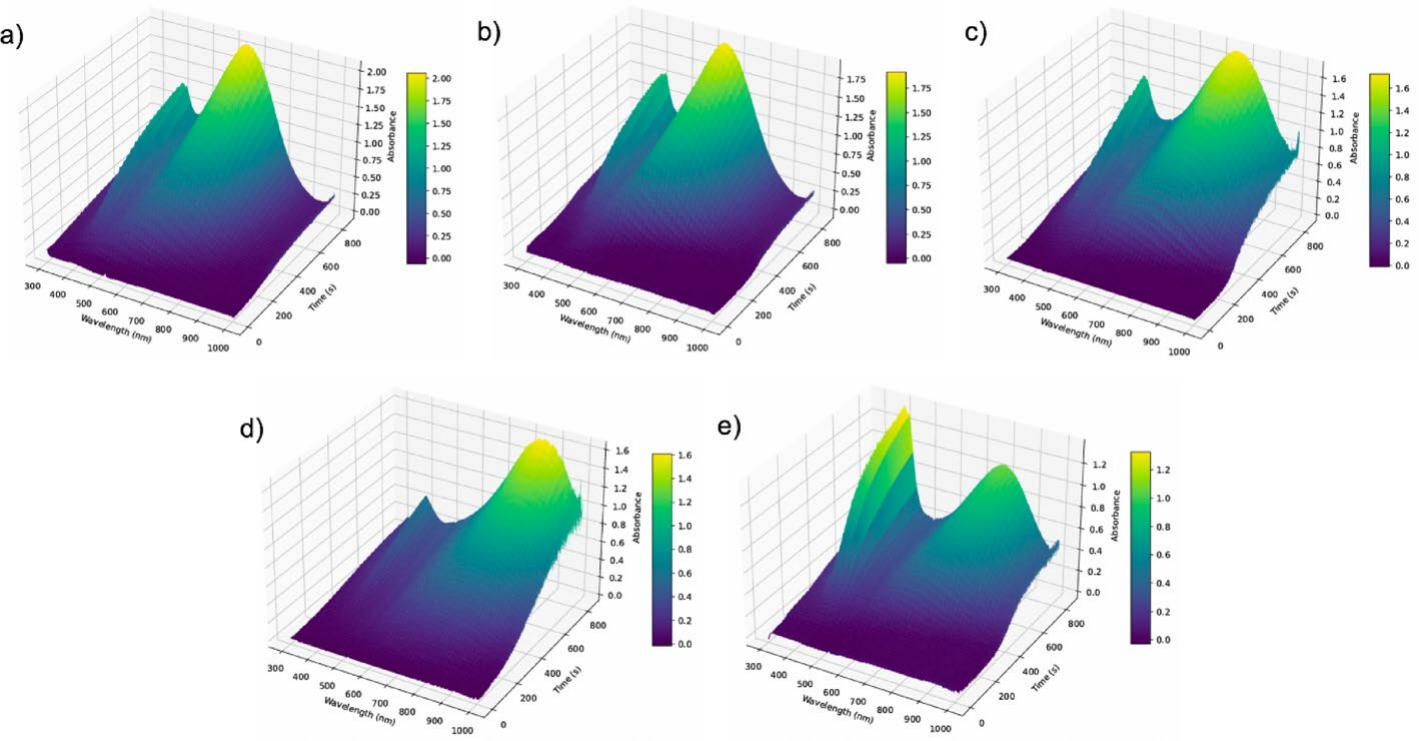
Transient UV–Vis spectra recorded during the growth step of silver nanoprisms synthesized with 70 µL seeds prepared using different citrate concentrations: **a** 30 mL; ** b** 35 mL; **c** 40 mL; **d** 45 mL; and **e** 50 mL

Analysis of the UV–Vis spectra and DLS distributions used as targets (Figs. 2 and 3) clearly shows that the citrate concentration employed during seed synthesis strongly influences the final morphology and optical absorption of the resulting particles. To further probe these effects, Fig. 5 presents the transient UV–Vis data collected during the growth step, starting from seeds generated with different citrate volumes. The transient traces reveal the progressive emergence of the two characteristic absorption peaks of silver nanoprisms; however, both the intensity and the spectral position of these features vary depending on the citrate concentration used during seed formation. These variations provide a diverse set of spectral-temporal signatures that were critical for training the CNN, enabling it to learn how subtle changes in seed quality translate into distinct growth dynamics and final particle morphologies.

**Fig. 6:**
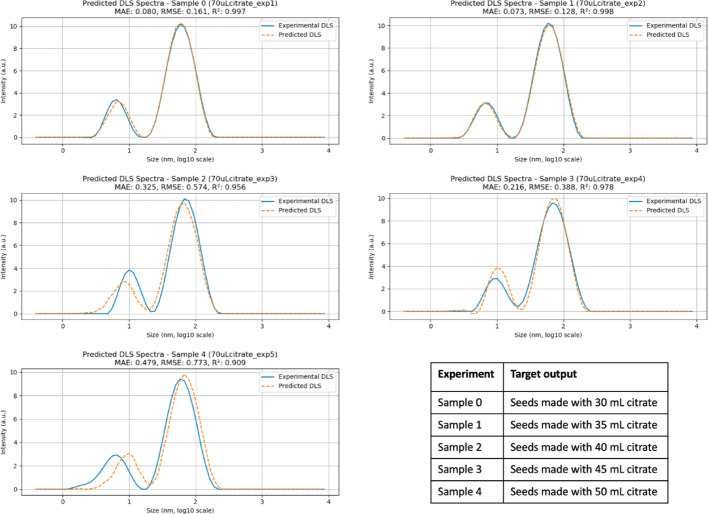
Predicted DLS values for samples prepared with 70 µL of seeds, using the proposed CNN architecture. Subpanels report MAE (nm), RMSE (nm), and R^2^ as performance metrics. The accompanying table specifies which experiment was used as the target output in each prediction

The proposed architecture was designed to predict the final DLS and UV–Vis spectra of a target experiment by processing the entire transient UV–Vis dataset. The results for the DLS prediction are presented in Fig. 6. Each subpanel includes the values of MAE, RMSE, and R^2^. The MAE, defined as the average absolute difference between predicted and true values, indicates how far the predictions deviate from the actual data. The RMSE, calculated as the square root of the average squared differences, provides similar information but penalizes larger errors more strongly. Finally, R^2^ quantifies how well the predictions approximate the experimental values.

Prediction fidelity was evaluated using MAE, RMSE, and R^2^ for the two characteristic DLS peaks centered at approximately 8 nm and 65 nm. For the highest-performing prediction cases, MAE values below 0.1 nm and RMSE values below 0.2 nm were obtained, corresponding to R^2^ values of approximately 0.99. These errors represent less than 2% of the smaller peak dimension and are negligible relative to typical DLS measurement variability, indicating excellent predictive accuracy.

Intermediate prediction performance was observed for MAE values around 0.32 nm and RMSE values near 0.57 nm, with a corresponding R^2^ of approximately 0.96. In the lowest-fidelity cases, MAE increased to approximately 0.48 nm and RMSE to 0.73 nm, accompanied by a reduction in R^2^ to approximately 0.90. Even in these cases, the errors remain small relative to the dominant particle size (~ 65 nm), supporting the robustness of the proposed CNN-based framework.

UV–Vis prediction fidelity was evaluated using Cosine similarity and Pearson correlation, which respectively quantify agreement in spectral shape and linear correlation of intensity variations. Values approaching unity indicate strong agreement between predicted and experimental spectra. In this study, Cosine similarity and Pearson correlation values above approximately 0.98 correspond to predictions that accurately reproduce the main plasmonic features, including peak position and overall spectral profile. Predictions with similarity values in the range of approximately 0.95–0.98 generally capture the dominant spectral trends but may exhibit deviations in relative peak intensity or bandwidth. Lower similarity values are associated with more pronounced discrepancies, such as peak shifts or changes in spectral curvature, which are also visually apparent in the corresponding spectra.

As with the DLS results, the first two predictions are nearly perfect, with both Cosine and Pearson values approaching 1. Prediction fidelity decreases in the subsequent cases, although the model still correctly identifies the number of UV–Vis absorption peaks and their relative positions. In particular, the last two predictions display statistical deviations from the expected outcomes. These deviations appear to arise either from an increased separation between absorption peaks (sample 3) or from changes in the peak intensity ratio (sample 4), where the CNN had fewer training examples.

Overall, the findings indicate that greater variability in the final spectra increases the data requirements, as is common in machine learning approaches. Nevertheless, as with the DLS predictions, and given the relatively limited number of experimental runs, the results can be considered highly representative of the actual outcomes. This demonstrates the validity of the approach for accelerating discovery, improving reproducibility, and standardizing materials synthesis protocols.

The predicted UV–Vis spectra occasionally appear noisier than the corresponding DLS predictions. This difference primarily reflects the nature of the prediction tasks rather than instability of the model. UV–Vis spectra are high-dimensional signals with closely spaced wavelength channels and inherently include experimental noise associated with in situ acquisition, flow conditions, and baseline fluctuations. In contrast, DLS predictions are low-dimensional and dominated by two characteristic size populations, resulting in visually smoother outputs.

**Fig. 7:**
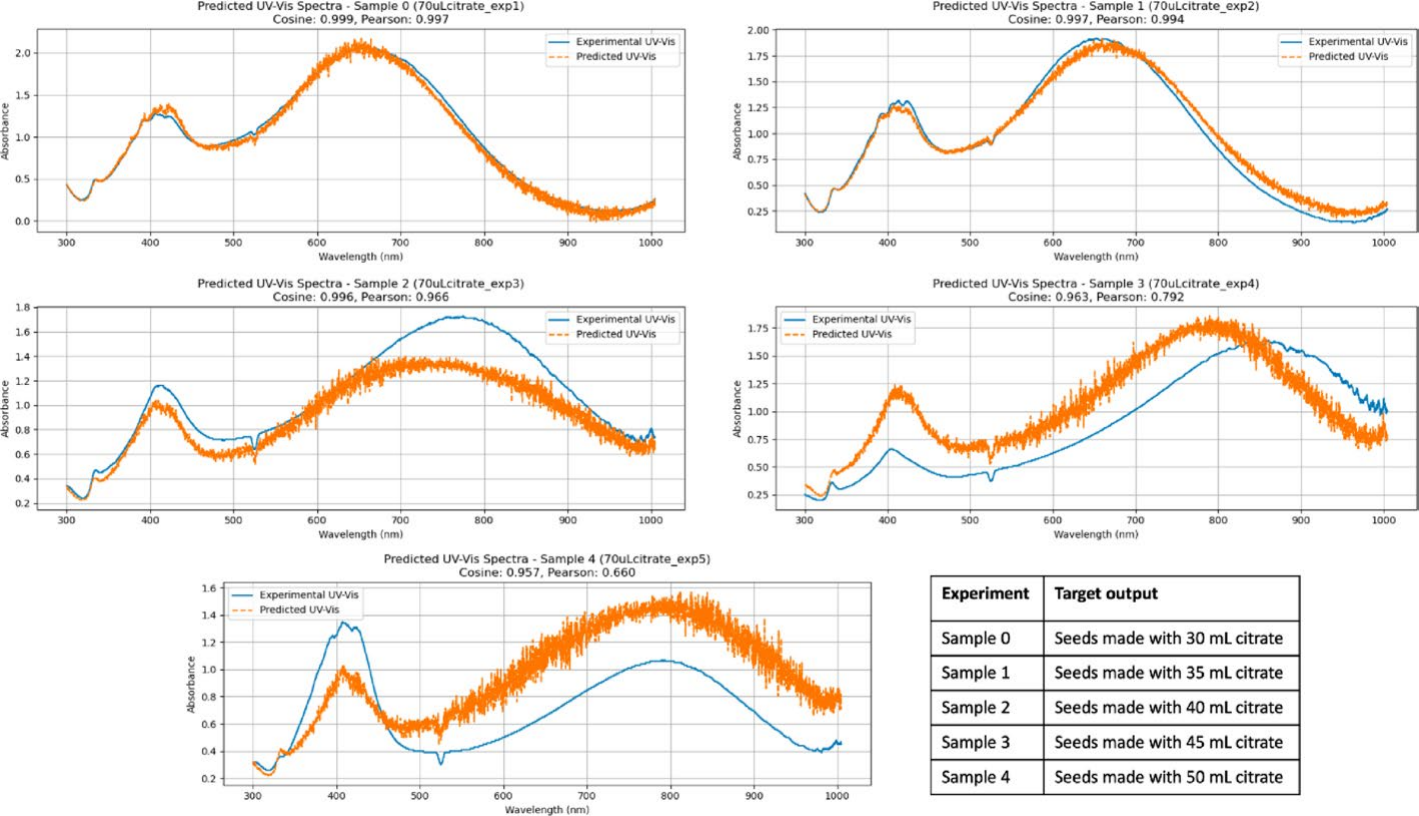
Predicted UV–Vis values for samples prepared with 70 µL of seeds, using the proposed CNN architecture. Cosine similarity and Pearson correlation values are reported to quantify agreement in spectral shape and intensity trends; deviations in these metrics correspond to visible differences in peak position or relative intensity. The accompanying table specifies which experiment was used as the target output in each prediction

Importantly, the CNN was trained to reproduce the full spectral intensity profile rather than smoothed peak parameters. As a result, small local deviations in intensity across adjacent wavelengths may appear as noise, even when the key plasmonic features, such as peak position, number of peaks, and overall spectral shape, are accurately captured. This behavior is quantitatively reflected in the high Cosine similarity and Pearson correlation values obtained for most predictions (Fig. 7).

**Fig. 8:**
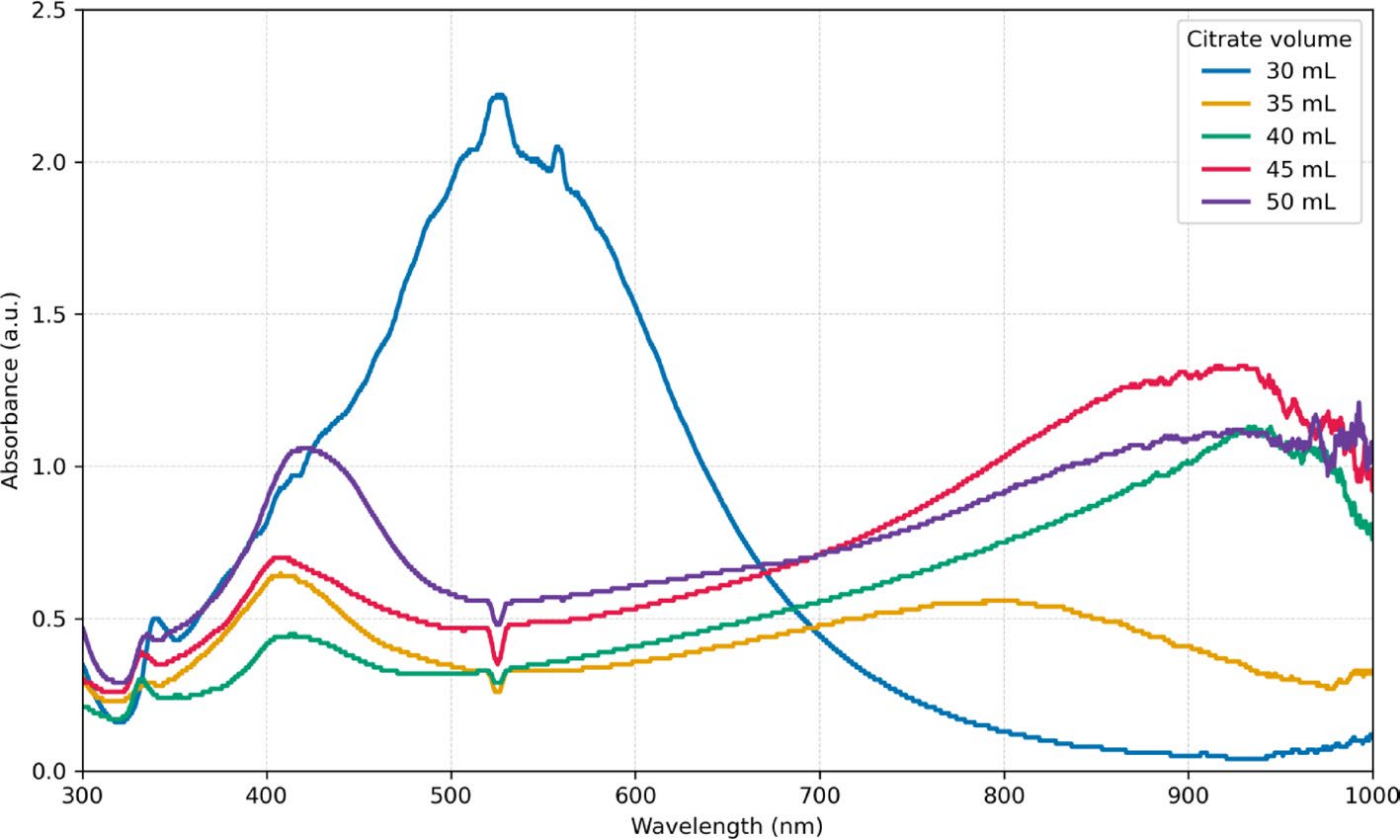
UV–Vis spectra of silver nanoprisms synthesized with 60 µL of seed solution. Seeds were generated using different citrate concentrations during the seed formation step, resulting in variations in the final optical response

**Fig. 9:**
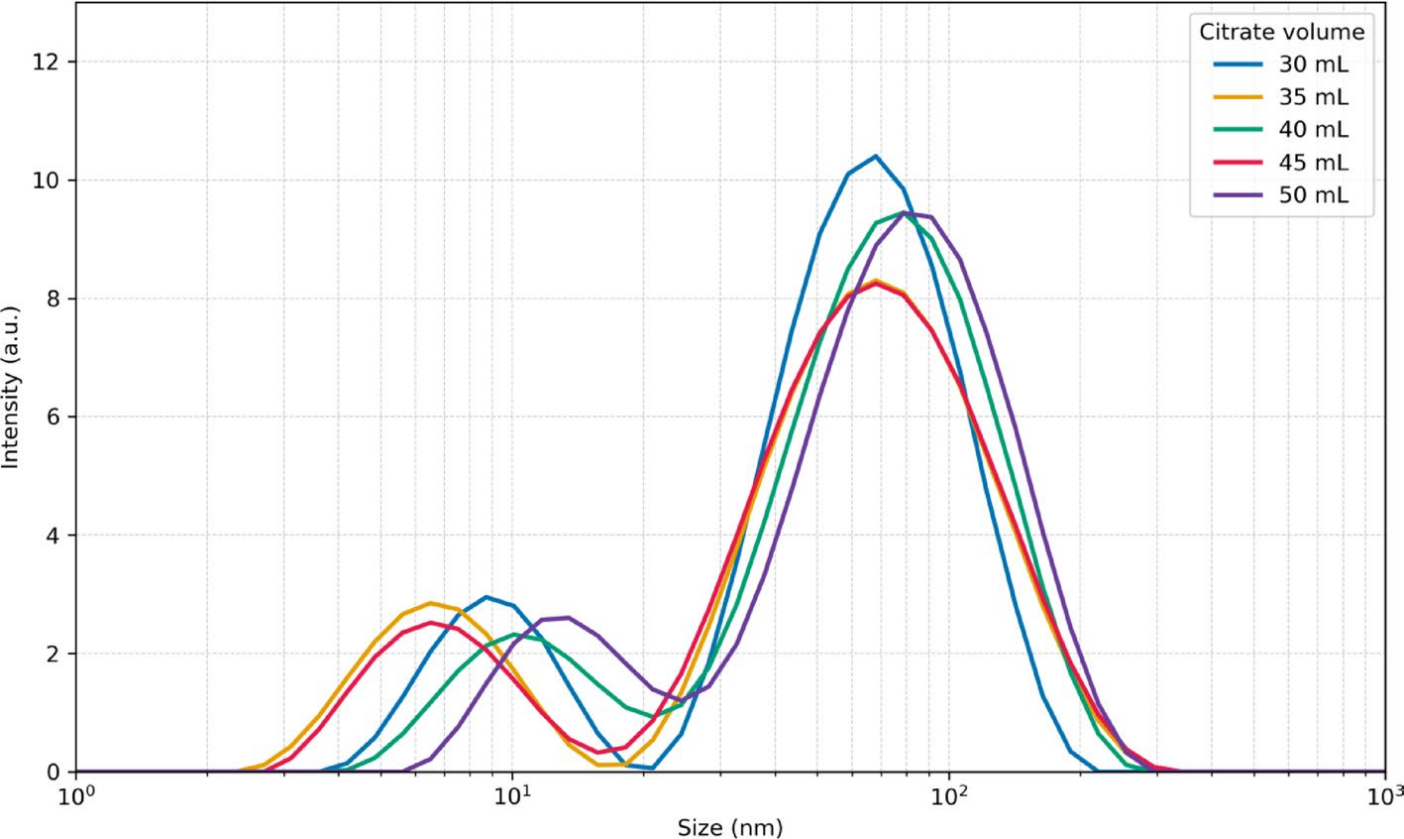
DLS size distributions of silver nanoprisms synthesized with 60 µL of seed solution. Seeds were generated using different citrate concentrations during the seed formation step, leading to distinct hydrodynamic size populations

For particles prepared from 70 µL of seeds, the final DLS and UV–Vis spectra displayed similar general features: both exhibited two peaks with comparable area ratios. This naturally raises the question of how the proposed approach performs when the spectral and size characteristics differ. To test this hypothesis, the same procedure was applied using 60 µL of seeds. According to Aherne et al. [11], this condition shifts the strongest absorption peak further to the red while preserving the smaller absorption band, consistent with an increase in nanoprism edge length while maintaining prism thickness. Figures 8 and 9 illustrate these effects: samples prepared with 60 µL of seeds and 40 mL citrate exhibit a central absorption at 950 nm (in contrast to ~ 750 nm for 70 µL seeds) and two DLS peaks, one near 10 nm (attributable to prism thickness and similar to that observed with 70 µL seeds) and another around 80 nm (compared to ~ 65 nm for 70 µL seeds).

**Fig. 10:**
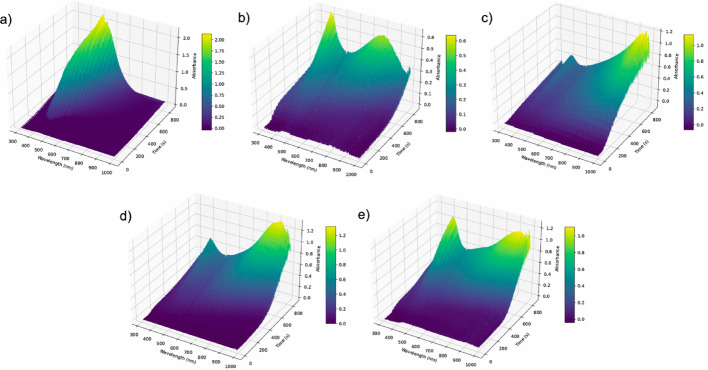
Transient UV–Vis spectra recorded during the growth step of silver nanoprisms synthesized with 60 µL seeds prepared using different citrate concentrations: **a** 30 mL; **b** 35 mL; **c** 40 mL; **d** 45 mL; and **e** 50 mL

For particles produced from 60 µL of seeds, the amount of citrate used during seed formation had a more pronounced effect on particle quality, influencing both optical absorption (Fig. 8) and morphology (Fig. 9). The transient UV–Vis data collected during particle growth also showed substantial differences (Fig. 10), which were subsequently used to train the CNN. This scenario provides an ideal test case for the hypothesis that maintaining spectral similarity is important for reducing the number of experimental runs required to effectively train the CNN.

**Fig. 11:**
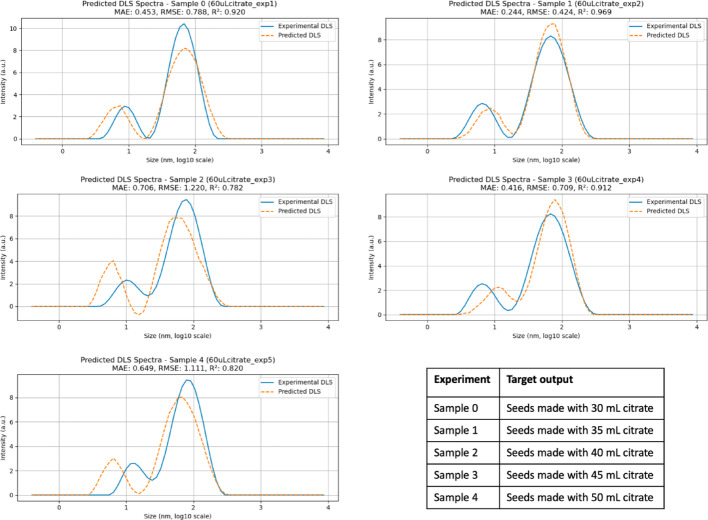
Predicted DLS values for samples prepared with 60 µL of seeds, using the proposed CNN architecture. Subpanels report MAE (nm), RMSE (nm), and R^2^ as performance metrics. The accompanying table specifies which experiment was used as the target output in each prediction

**Fig. 12:**
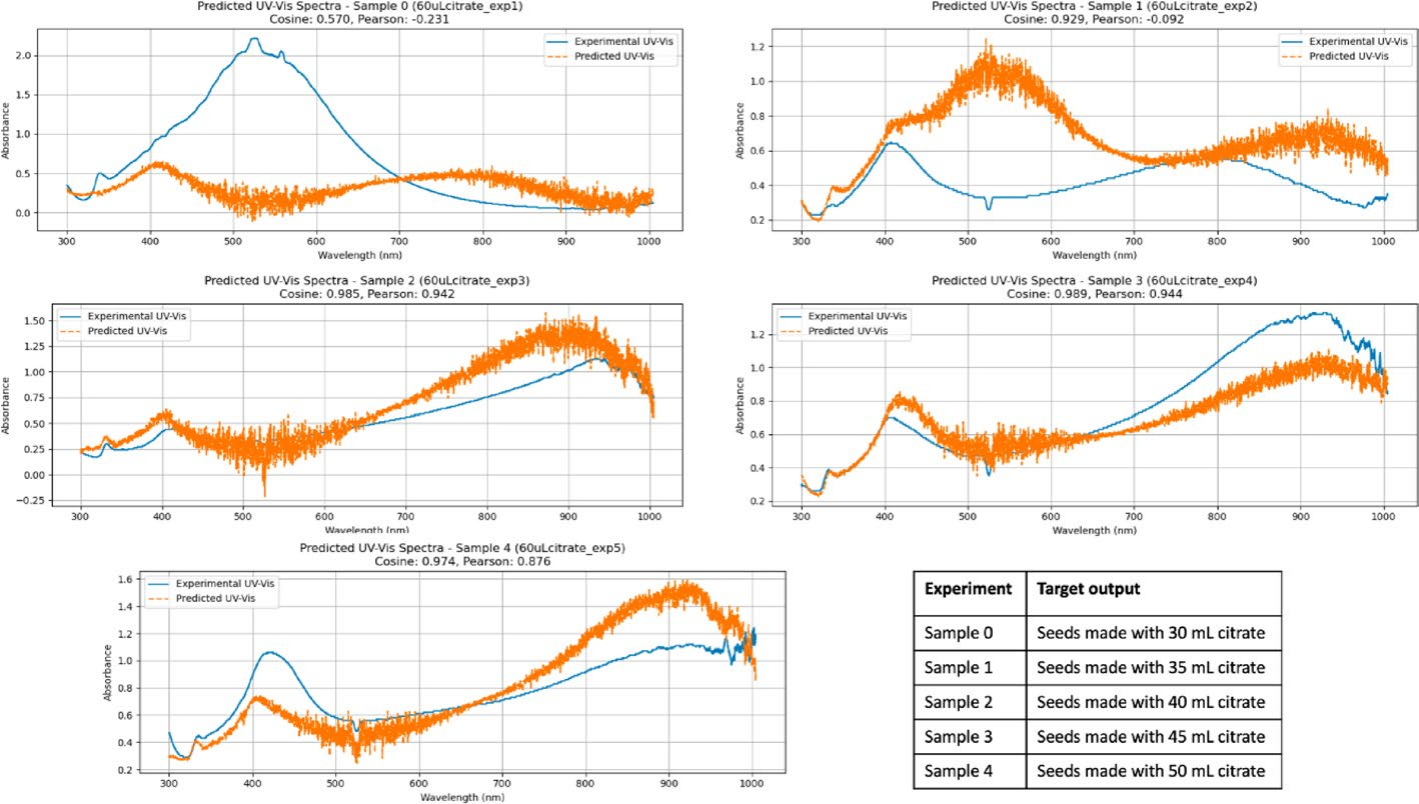
Predicted UV–Vis values for samples prepared with 60 µL of seeds, using the proposed CNN architecture. Cosine similarity and Pearson correlation values are reported to quantify agreement in spectral shape and intensity trends; deviations in these metrics correspond to visible differences in peak position or relative intensity. The accompanying table specifies which experiment was used as the target output in each prediction

The prediction outcomes are presented in Figs. 11 and 12. Using the same number of training data points as for particles prepared with 70 µL of seeds results in reduced prediction fidelity for the 60 µL condition. This reduction does not reflect an inherent limitation of the CNN in handling increased spectral variability. Instead, the 60 µL seed condition exhibits substantially higher variability in both the transient UV–Vis spectra and the final particle size distributions, leading to a broader dispersion of outcomes for a fixed number of training examples. Under such circumstances, a larger dataset is required to adequately sample the accessible synthesis space and achieve balanced predictions. This limitation is particularly evident in the UV–Vis predictions when the target spectrum differs markedly from the others, for example for samples prepared with seeds formed using 30 or 35 mL of citrate.

Importantly, the CNN was trained independently for each seed volume using datasets generated within the same synthesis regime. The predictions shown for the 60 µL condition therefore do not represent extrapolation from the 70 µL dataset, but rather reflect model performance within a locally defined parameter space. The present framework is thus intended to support protocol optimization and refinement under controlled parameter variations, as commonly encountered during synthesis scale-up, rather than to predict outcomes under extreme extrapolation far outside the training domain.

The increased apparent noise in some of the predicted UV–Vis spectra for the 60 µL condition follows the same origin discussed above for the 70 µL case and reflects the higher dimensionality and variability of the spectral data rather than reduced model stability.

Notably, the increased variability observed for the 60 µL seed condition does not follow a simple monotonic or systematic trend when compared to neighboring synthesis protocols. Whereas samples prepared with 70 µL of seeds yield relatively consistent optical and morphological features, the 60 µL condition displays pronounced dispersion across citrate concentrations. At present, this behavior cannot be straightforwardly rationalized in terms of classical nucleation or growth mechanisms and is therefore treated as a representative example of a high-variability synthesis space.

Importantly, this case illustrates a general principle relevant to machine-learning-assisted synthesis: as experimental variability increases, so does the minimum number of experiments require for robust model training. The use of transient, in situ UV–Vis data partially mitigates this requirement by enriching each experiment with time-resolved information, thereby reducing, but not eliminating, the need for additional experimental runs. Despite the reduced fidelity, several key spectral features are still accurately reproduced, suggesting that the necessary increase in training data does not need to be excessive. This is encouraging for labor-intensive synthesis protocols that are traditionally optimized through trial-and-error approaches.

We note that formal uncertainty quantification, such as prediction intervals or confidence bounds, is not included in the present study. Meaningful uncertainty estimation for deep learning models typically relies on ensemble-based approaches, Bayesian approximations, or probabilistic modeling frameworks, which generally require substantially larger datasets to yield statistically reliable estimates [[27, 28]]. In data-scarce experimental regimes such as nanomaterial synthesis, uncertainty quantification remains an open research challenge, as withholding additional data for validation or calibration can significantly degrade model training and bias uncertainty estimates. Given the intentionally limited size of the experimentally generated dataset used here, we therefore refrain from presenting formal uncertainty measures that could be misleading or poorly constrained.

Instead, predictive fidelity is evaluated using established and widely adopted performance metrics, including MAE, RMSE, and the R^2^ for DLS predictions, as well as Cosine similarity and Pearson correlation for UV–Vis spectral predictions. These metrics are commonly used in both machine-learning and materials science literature to provide robust and interpretable measures of agreement when dataset size limits more elaborate statistical characterization [[29, 30]]. Together, they allow reliable assessment of model performance in terms of absolute error, variance explained, and spectral similarity, which is appropriate for the scope and objectives of the present proof-of-concept study. Incorporating uncertainty-aware modeling strategies represents an important direction for future work as larger experimental datasets become available.

## Concluding remarks and outlook

In summary, we demonstrate a CNN-assisted, data-efficient framework for predicting the morphology and optical properties of silver nanoprisms directly from in situ transient UV–Vis data. By embedding spectral-temporal information into model training, the approach captures growth dynamics that are inaccessible to conventional endpoint analyses, enabling predictive control of plasmonic responses with a limited number of experiments.

While model explainability tools such as saliency maps or SHAP analysis offer promising routes for interpreting neural network predictions, their application to noisy, high-dimensional spectro-temporal data remains non-trivial and requires careful methodological validation. We therefore avoid presenting potentially misleading interpretability visualizations at this stage and identify explainability analysis as an important direction for future work as larger datasets become available.

The present study establishes a proof-of-concept strategy for integrating machine learning into nanomaterial synthesis, shifting optimization from empirical trial-and-error toward more rational, data-guided protocol design. Although demonstrated here for silver nanoprisms, the underlying framework is expected to be adaptable to other plasmonic and nanostructured materials where morphology- function relationships are governed by dynamic growth processes. Looking forward, coupling such models with continuous-flow synthesis and adaptive feedback loops may enable autonomous, scalable, and reproducible development of nanomaterials for photonic, sensing, and catalytic applications.

## Data Availability

All experimental datasets and trained model configurations used in this study are available from the corresponding author upon reasonable request.
